# Supplementary material for: ATP8B1 Deficiency Results in Elevated Mitochondrial Phosphatidylethanolamine Levels and Increased Mitochondrial Oxidative Phosphorylation in Human Hepatoma Cells
Source: Int J Mol Sci. 2022 Oct 15;23(20):12344. doi: 10.3390/ijms232012344 (PMC9604224; doi:10.3390/ijms232012344)
Supplement: Supplementary file 1 [file ijms-23-12344-s001.zip › ijms-1893430-supplementary.pdf]

Supplementary figures/tables and statements and declarations

Figure S1:

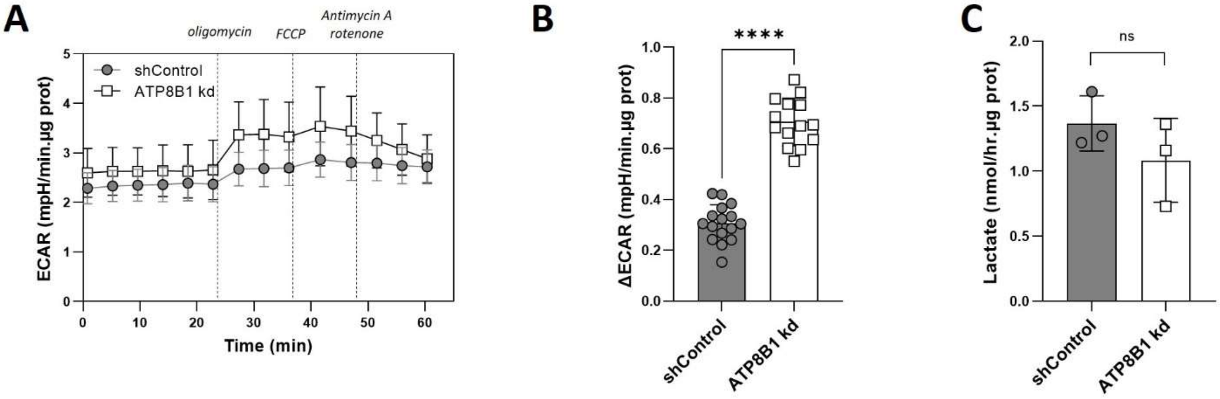

Figure S1

(A) Extracellular acidification rate under 5.5 mM glucose condition. Dotted lines indicate the addition of different compounds to modulate mitochondrial respiration. Profiles shown are means +/- s.d. of 14-16 replicates from 2 independent experiments.

(B) Quantification of oligomycin-induced (ATP-coupled) ECAR in the presence of glucose. Data are extracted from the data shown in (A) and are expressed as means +/- s.d. of 14-16 replicates of 2 independent experiments; ****p<0.0001 by unpaired t.test.

(C) Lactate measurement in the medium under identical conditions as in (A). Lactate concentration is expressed as means of 18 replicates of 2 independent experiments +/- standard deviation. Statistical analysis by an unpaired t.test.

Figure S2:

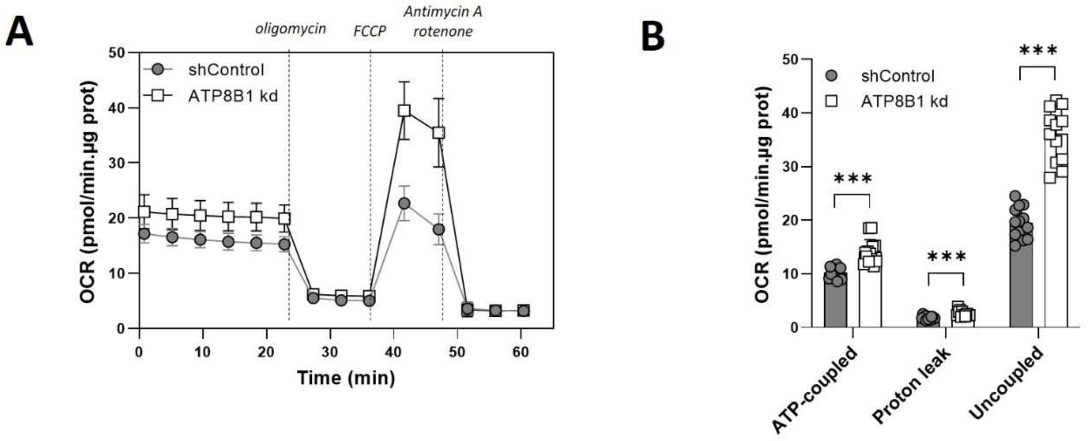

Figure S2

(A) Oxygen consumption rate (OCR) in the absence of glucose and presence of 125 µM octanoate. Profiles shown are means +/- s.d. of 13 replicates of 2 independent experiments.

(B) Quantification of ATP-coupled-, leak-associated and uncoupled OCR in the presence of octanoate. Data are expressed as means +/- s.d. of 13 replicates of 2 independent experiments; ***$p<0.001$, multiple unpaired t.test.

Figure S3:

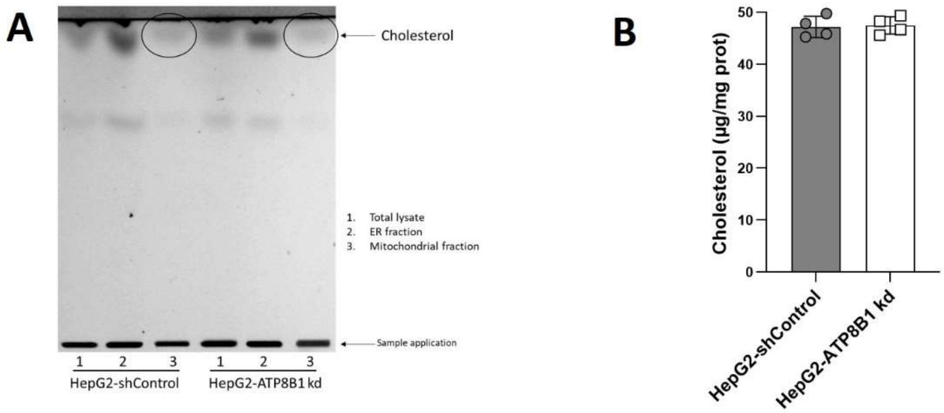

Figure S3 Cholesterol content of mitochondria from control and ATP8B1 knockdown cells. Shown are data from 2 independent experiments.

(A) In our analysis of mitochondrial lipid species (figure 5A) we also analysed a mitochondria isolation on HPTLC for cholesterol abundance. Samples were processed as described in Material and Methods and separated using chloroform/ethylacetate (3:7) as running buffer. The encircled bands indicate mitochondrial cholesterol.

(B) Quantification of mitochondrial cholesterol content in HepG2 cells. Mitochondria were isolated and lipids were extracted as described in Material and Methods. Total cholesterol was enzymatically determined with the Amplex Red Assay Kit (Thermo Scientific) according to manufacturer instructions. Fluorescence was measured using a CLARIOstar monochromator microplate reader (BMG LABTECH). Shown are 4 biological replicates from 1 mitochondria isolation corrected for mitochondrial protein content.

Figure S4:

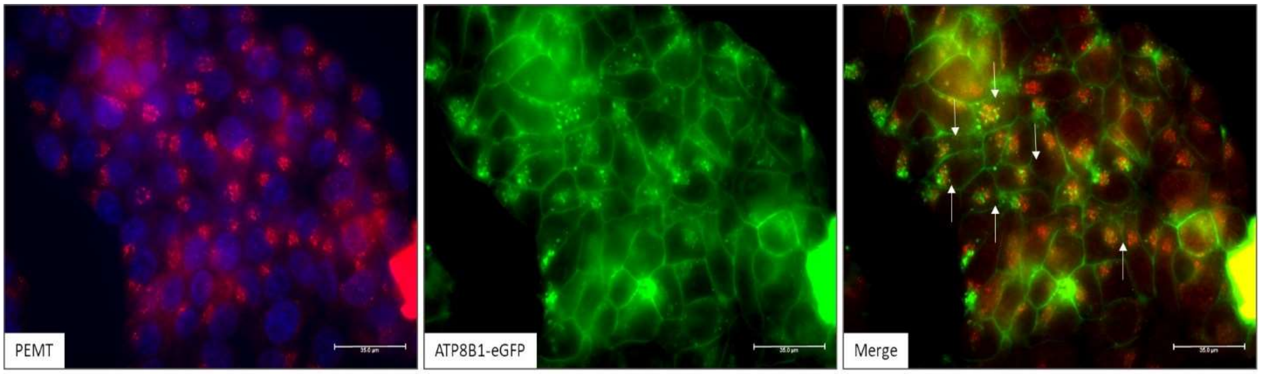

Figure S4
Immunofluorescent detection of PEMT (red), ATP8B1-eGFP (green) and nuclear dapi in blue. Arrows indicate areas where ATP8B1-eGFP and PEMT punctae are in close proximity. Bar = 35 nm.

Figure S5:

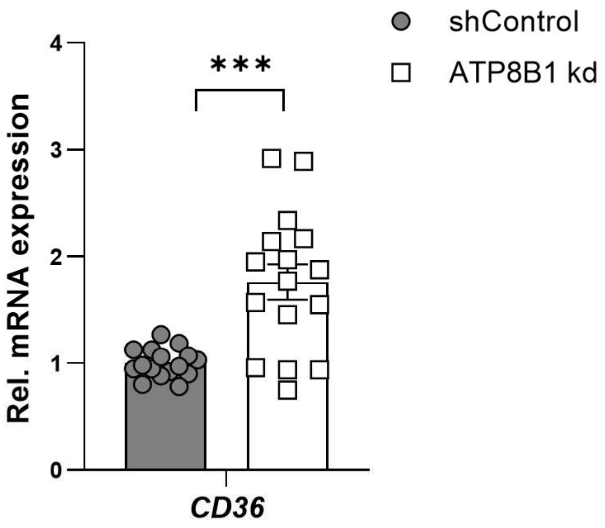

Figure S5
CD36 mRNA expression in ATP8B1 knockdown HepG2 cells. Data were normalized to shControl cells and are expressed as means +/- s.e.m. of 16 replicates from 5 independent experiments. Statistical analysis by an unpaired t.test. ***p<0.0001.

Table S1. Sequences of primers used

Target	**Forward sequence (5’-3’)**	**Reverse sequence (5’-3’)**
ATP8B1	TGGTGGATAGGACTGATGGTC	CGTTTACCAGGGCACCTTC
LDLR	GTGTCACAGCGGCGAATG	CGCACTCTTTGATGGGTTCA
PEMT	CTGGAATGTGGTTGCACGATG	GCTTAGAGAGTAGCAGGCCA
RPLP0 (36B4)	TCATCAACGGGTACAAACGA	GCCTTGACCTTTTCAGCAAG
Cyclophilin	ACGGCGAGCCCTTGG	TTTCTGCTGTCTTTGGGACCT
HPRT	CCTGGCGTCGTGATTAGTGAT	AGACGTTCAGTCCTGTCCATAA
MT-ND1	AGCCTAGCCGTTTACTCAATCCT	GTGTTGTGATAAGGGTGGAGAGG
ADCY10	CACTTGAAATGTGCCCGCTT	CATGTCTAAAGCGTTGAGCCG

Statements and declarations

Funding

This research received no external funding. Valentina E. Gómez-Mellado was funded by a Tytgat Institute grant.

Acknowledgements

Not applicable

Author contributions

Conceptualization, Valentina Gómez-Mellado, Ronald Oude-Elferink, Arthur Verhoeven and Coen Paulusma; Data curation, Carmen Bernardino Morcillo, Remco Kersten and Coen Paulusma; Formal analysis, Valentina Gómez-Mellado, Jung-Chin Chang, Arthur Verhoeven and Coen Paulusma; Investigation, Valentina Gómez-Mellado, Jung-Chin Chang, Kam Ho-Mok, Carmen Bernardino Morcillo, Remco Kersten, Arthur Verhoeven and Coen Paulusma; Methodology, Valentina Gómez-Mellado, Jung-Chin Chang, Kam Ho-Mok, Ronald Oude-Elferink, Arthur Verhoeven and Coen Paulusma; Project administration, Coen Paulusma; Resources, Ronald Oude-Elferink; Supervision, Coen Paulusma; Validation, Valentina Gómez-Mellado, Jung-Chin Chang, Kam Ho-Mok, Ronald Oude-Elferink, Arthur Verhoeven and Coen Paulusma; Visualization, Valentina Gómez-Mellado, Remco Kersten, Arthur Verhoeven and Coen Paulusma; Writing – original draft, Valentina Gómez-Mellado and Coen Paulusma; Writing – review & editing, Ronald Oude-Elferink, Arthur Verhoeven and Coen Paulusma.
All authors will be informed about each step of manuscript processing including submission, revision, revision reminder, etc. via emails from our system or assigned Assistant Editor.

*

☑ All listed authors meet the ICMJE criteria and all who meet the four criteria are identified as authors. We attest that all authors contributed significantly to the creation of this manuscript, each having fulfilled criteria as established by the ICMJE.

*

☑ We confirm that the manuscript has been read and approved by all named authors.

*

☑ We confirm that the order of authors listed in the manuscript has been approved by all named authors.

Institutional Review Board Statement

Not applicable

Informed consent statement

Not applicable

Data availability statement

The raw datasets generated and/or analysed during the current study are available from the corresponding author on reasonable request.

Ethics approval and consent to participate

Not applicable

Conflicts of interests

The authors have no relevant financial or non-financial interests to disclose

Abbreviations

PFIC, progressive familial intrahepatic cholestasis; OXPHOS, oxidative phosphorylation; ETC, electron transport chain; LDLR, low-density lipoprotein receptor; PEMT, phosphatidylethanolamine N-methyltransferase; DCA, dichloroacetic acid.
